# Orbital reconstruction: a systematic review and meta-analysis evaluating the role of patient-specific implants

**DOI:** 10.1007/s10006-022-01074-x

**Published:** 2022-05-20

**Authors:** Sanjeev Kotecha, Ashley Ferro, Patrick Harrison, Kathleen Fan

**Affiliations:** 1 Oral and Maxillofacial Surgery Department, King’s College Hospital NHS Foundation Trust, London, UK; 2 Faculty of Dentistry, Oral and Craniofacial Sciences, King’s College London, UK

**Keywords:** Orbital fractures, Orbital implants, Reconstructive surgical procedures, Maxillofacial injuries, Stereolithography

## Abstract

The purpose of this study is to execute an evidence-based review answering the following question (PICO): “Do patient-specific implants (PSI), manufactured or designed using computer-assisted technology, improve outcomes (orbital volume change, enophthalmos, diplopia, and operative duration) compared to conventional methods in orbital reconstruction following traumatic orbital injury in the adult patient population?” We performed a systematic review and meta-analysis in accordance with PRISMA guidelines. Inclusion criteria included any comparative paper whereby computer-assisted technology was used in the prefabrication or design process of implants for use in post-traumatic orbital reconstruction. Paediatric patient populations were excluded. Eight databases were systematically searched for relevant studies. Risk of bias was assessed through the NOS and RoB2 tools. Random-effects models were used to identify differences in outcomes between groups where possible. Analysis was performed using R 4.0.0. Eleven of 4784 identified studies were included, comprising 628 adult patients, with 302 and 326 patients in the patient-specific and conventional groups, respectively. Weighted mean difference between unaffected and post-operative orbital volume was 0.32 ml (SD 0.75) and 0.95 ml (SD 1.03) for patient-specific and conventional groups, respectively. Significant improvement was identified in post-operative orbital volume reconstitution with the use of PSI, compared to conventional implants, in 3 of the 5 reporting studies. Equally, post-operative enophthalmos trended towards lower severity in the patient-specific group, with 11.2% of patients affected in the patient-specific group and 19.2% in the conventional group, and operative duration was significantly reduced with the use of PSI in 3 of the 6 reporting studies. Despite a tendency to favour PSI, no statistically significant differences in key outcomes were identified on meta-analysis. Although there is some encouraging data to support improved outcomes with the use of patient-specific orbital implants in post-traumatic reconstruction, there is, at present, no statistically significant evidence to objectively support their use over conventional implants based on the currently available comparative studies. Based on the results of this study, the choice of implant used should, thus, be left to the discretion of the surgeon.

**Supplementary Information:**

The online version contains supplementary material available at 10.1007/s10006-022-01074-x.

## Introduction

Orbital fractures are commonplace in craniomaxillofacial trauma, accounting for approximately 10–25% of injuries to the region [1, 2]. Management of these injuries is inherently challenging as clinical sequelae, both functional and aesthetic, may not always be immediately obvious. Thus, a period of observation may be sensible in the acute setting. Inappropriate management, however, may result in reduced visual acuity, persistent enophthalmos, ocular motility deficits, diplopia, and sensory disturbance [3]. Operative management to repair the defect may be warranted either immediately (such as for trapdoor fractures with entrapment in paediatric patients or in the case of a profound oculocardiac reflex with the possibility of haemodynamic instability) or delayed, according to persistence or progression of symptoms post-trauma [4, 5]. Indeed, indications quoted for delayed operative management, ideally within 2 weeks of the inciting trauma, include enophthalmos (> 2 mm); ocular dysmotility; persistent diplopia; computed tomography (CT) findings of extraocular muscle entrapment; progressive infraorbital nerve (ION) hypoaesthesia; and abnormal forced duction testing [6].

Orbital reconstruction following trauma is complicated by the limited operative view and the complexity of the anatomical region with the presence of vital neurovasculature in close proximity [7]. The procedure involves mobilisation of entrapped soft tissues and the restoration of the orbit to its correct anatomical position and volume by replacing the bony defect and providing stable fixation using an implant [8, 9]. The decision on which implant material to use is influenced by surgeon experience, fracture severity, patient characteristics, and cost; traditionally, conventional implants have taken the form of either prefabricated (i.e. mass-produced) alloplastic plates, such as titanium, or autologous bone grafts [10]. Conventional reconstruction methods afford the advantages of availability and biostability allows intra-operative contouring as well as cost-effectiveness. The emergence and widespread availability of computer-assisted surgery (CAS), including computer-aided design and computer-aided manufacturing (CAD/CAM) processes, has precipitated the development of individualised implants. These may take one of two forms: either entirely bespoke implants which are usually manufactured externally following provision of a CT scan–derived mirror-image overlay (MIO) from the contralateral, uninjured orbit (hereafter termed patient-specific implants (PSI)); or conventional implants that are manipulated and contoured pre-operatively on patient-specific, three-dimensional (3D) models created through stereolithography from CT imaging (hereafter termed hybrid-PSI) [11]. The perceived hypothetical benefits of using PSI or hybrid-PSI in orbital reconstruction are apparent and include reduced operative time (through avoiding the need for intra-operative contouring) and more precise reconstitution of the patient’s pre-morbid orbital architecture to optimise restoration of both function and form. Nonetheless, despite the promise of patient-specific implants, there is limited comparative data in the literature supporting their benefit over conventional implants. Previous systematic reviews on their use in orbital reconstruction have either included heterogeneous datasets (such as defects secondary to neoplasia and resection, thereby precluding a formal quantitative comparison of key outcomes) or featured incomplete search strategies, such that key comparative studies were not included [12]. Moreover, although comprehensive reviews have been conducted on the use of computer-assisted, technology-augmented surgery in post-traumatic orbital reconstruction, no study to the authors’ knowledge has thus far provided a quantitative evaluation of relevant clinical outcomes between conventional reconstruction methods and PSI [8]. The purpose of the current systematic review and meta-analysis was, therefore, to synthesise the currently available data from comparative studies on patient-specific (hereafter encompassing both PSI and hybrid-PSI) implants vs. conventional reconstruction methods with respect to operative time, enophthalmos, diplopia, and orbital volume reconstitution.

## Materials and methods

This study was completed in keeping with the Preferred Reporting Items for Systematic Reviews and Meta-Analyses (PRISMA) guidelines. Institutional review board (IRB) approval was not required for the current study.

### Research question (according to the PICO framework)

Do patient-specific implants, manufactured or designed using computer-assisted technology, improve outcomes (orbital volume change, enophthalmos, diplopia, and operative duration) compared to conventional methods in orbital reconstruction following traumatic orbital injury in the adult patient population?

### Search strategy

A systematic literature search was performed on 24 January 2022 using the following databases: ClinicalTrials.gov; Cochrane CENTRAL; EMBASE via OVID; MEDLINE via OVID; PubMed; Scopus; Web of Science Core Collection; and World Health Organization International Clinical Trials Registry Platform. The detailed search strategy is appended in the Supplementary Material S1. References of identified records were iteratively searched for further suitable records.

### Selection criteria

#### Inclusion criteria:

1. Computer-assisted technology in the prefabrication or design process of implants for use in orbital reconstruction following orbital fracture.
2. Implants were patient-specific, achieved either through bespoke fabrication based on CAD/CAM technologies (PSI) or through pre-operative bending of plates using patient-specific 3D modelling (hybrid-PSI).
3. Performed primary and/or secondary reconstruction of orbital fractures.
4. Provided details on at least one of the following outcomes: orbital volume change; enophthalmos; diplopia; hypoglobus; restricted ocular motility; ION hypoaesthesia; or procedure-related complications.

#### Exclusion criteria:

1. Computer-assisted technologies were used only for diagnostic, pre-operative planning, or intra-operative navigation purposes.
2. Observational studies only, including a single, non-comparative treatment arm.
3. Insufficient description of post-operative outcomes.
4. Orbital reconstruction performed for non-traumatic indications.

### Data collection

Titles, abstracts, and full texts were independently assessed by two reviewers (AF and SK). Discrepancies were resolved by consensus following discussion between reviewers to minimise selection bias. In addition to the exclusion criteria above, the following identified studies were excluded: case reports; review articles; recommendations and guidelines; expert opinions; full texts not available in English; cadaveric studies; animal studies; surveys; and shared data. A custom data collection form was used to extract the following data: study title; authors; year of publication; the nature of the study; randomisation method; patient demographics; mechanism of injury; time from injury to surgery; sample size of both patient-specific and conventional cohorts; implant type; manufacturing process; presence or absence of intra-operative navigation; fracture characteristics; surgical approach; whether reconstruction was primary or secondary; and follow-up duration. Outcomes measured were as follows: operative duration; enophthalmos (as measured using an exophthalmometer); diplopia; orbital volume change; hypoglobus; restriction of ocular motility; ION hypoaesthesia; and procedure-related complications. Where multiple follow-up periods were reported, data were extracted from the most recent follow-up.

### Assessment of bias

Risk of bias for the relevant studies was evaluated using two tools: the Newcastle–Ottawa Scale (NOS) was utilised for non-randomised studies and the Revised Cochrane Risk-of-Bias Tool for randomised trials (RoB2), both of which are recommended by the Cochrane Collaboration. Studies were not excluded on the grounds of bias. Instead, bias was acknowledged and highlighted. Non-randomised studies assessed using the NOS were classified as either high (score 0–3), moderate (score 4–6), or low risk of bias (score 7–9) based on evaluations of three domains [13]. Randomised studies assessed using RoB2 were given an overall ranking of low risk of bias, some concerns, or high risk of bias based on evaluations of five domains [14]. Discrepancies in scoring were resolved through consensus.

### Statistical analysis

Continuous outcomes and baseline characteristics, where available, were reported as weighted combined means using formulae provided by the Cochrane Collaboration [15]. Means and standard deviations were approximated according to Wan et al. (2014) where not otherwise provided [8]. Study heterogeneity was assessed using the Cochrane Q-statistics chi-square test and Higgins *I*^2^ statistic. *I*^2^ was interpreted as follows: 0–40% as low heterogeneity; 30–60% as moderate heterogeneity; 50–90% as substantial heterogeneity; and > 75% as considerable heterogeneity [15]. A Cochrane Q statistic *p*-value < 0.10 was considered significant. Meta-analysis of binary outcomes (complication vs. no complication, for example) was achieved using the Mantel–Haenszel approach as part of a random-effects model. Results are reported as risk ratios (RR) with 95% confidence intervals. For continuous outcomes (such as enophthalmos), Hedges’ *g* was used as the measure of standardised mean difference (SMD), and a random-effects model was performed using the Hartung-Knapp adjustment. For descriptive summary statistics, where significance between groups was not reported in identified studies, Welch’s two-tailed independent samples *t*-test was employed based on estimated means and standard deviations for continuous data and Fisher’s exact test for categorical data, where appropriate. Statistical analysis was performed using R Statistical Software version 4.0.0.

## Results

### Study selection

**Fig. 1:**
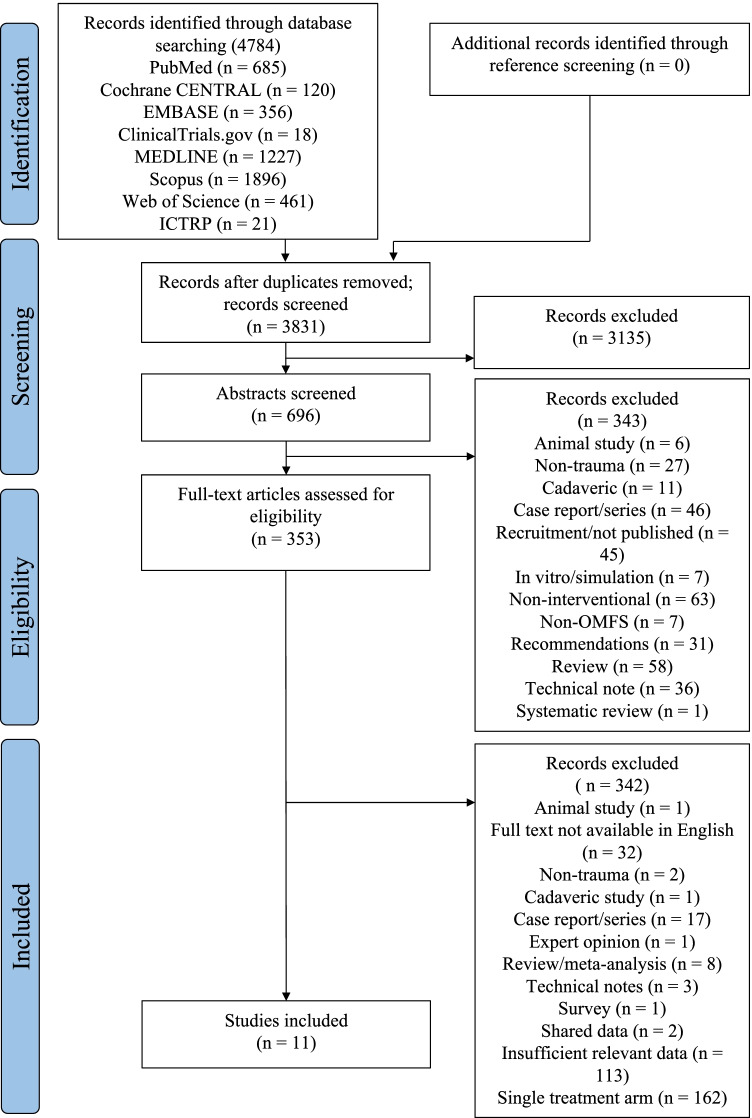
PRISMA flow diagram for study screening and selection

The initial literature search identified 3831 unique records across the 8 databases (Fig. 1) after removal of duplicates. Of these, 11 studies met the inclusion criteria for further analysis [16–26].

The NOS and RoB2 Tools were employed to assess risk of bias for the 9 non-randomised (scores ranged from 5 to 8) and 2 randomised studies (both evaluated as having some concerns), respectively. None of the 11 total studies was adjudged as having a high risk of bias (Tables 1 and 2).

**Table 1:** Newcastle–Ottawa Scale (NOS) total scores and ratings for risk of bias for the 7 observational studies; scores in the ranges of 0–3, 4–6, and 7–9 represent high, moderate, and low risk of bias respectively

Author	Selection (0–4)	Comparability (0–2)	Outcome (0–3)	Total NOS (0–9)	Overall risk of bias judgement
Fan et al	3	1	1	5	Moderate
Guo et al	3	0	3	6	Moderate
Khomutinnikova et al	3	1	2	6	Moderate
Kim et al	3	2	3	8	Low
Nkenke et al	3	2	3	8	Low
Scolozzi et al	3	1	3	7	Low
Sigron et al	3	1	1	5	Moderate
Timoshchuk et al	3	1	3	7	Low
Zimmerer et al	3	2	2	7	Low

**Table 2:** Revised Cochrane Risk-of-Bias Tool for Randomised Trials (RoB2); each of the 5 domains is rated as ‘low,’ ‘some concerns,’ or ‘high’ with an overall risk of bias awarded using the same ratings

Author	Domain 1 (randomisation process)	Domain 2 (deviations from intended interventions)	Domain 3 (missing outcome data)	Domain 4 (measurement of outcome)	Domain 5 (selection of reported result)	Overall risk of bias judgement
Gupta et al	Some concerns	Low	Some concern	Some concerns	Low	Some concerns
Raisian et al	Some concerns	Low	Low	Low	Some concerns	Some concerns

### Study characteristics

Of the 11 identified studies, 2 were randomised-controlled trials [18, 21], 6 were retrospective cohort studies [16, 17, 19, 23, 25, 26], and the remaining 3 were prospective cohort studies [20, 22, 24]. Characteristics of included studies are provided in Table 3.

**Table 3:** Overview of included studies. *NR*, not reported; *IPV*, interpersonal violence; *RTC*, road traffic collision

Study	Year	Sample size conventional	Sample size patient-specific	Patient age; years (SD)	Patient sex (F:M)	Mechanism of injury (frequency)	Primary vs. secondary	Approach	Implant type—conventional	Implant type—patient-specific
Fan et al	2017	27	29	36.6 (NR)	13:43	IPV 32 RTC 3 Fall 9 Sport-accident 10 Other 2	Primary	Transcaruncular and/or inferior fornix	MEDPOR TITAN Orbital floor implant	MEDPOR TITAN Orbital floor implant – 3D printed (manufacturer not disclosed)
Guo et al	2009	26	35	38.1 (10.2)	25:36	IPV 15 RTC 36 Falls 6 Sport-accident 4	Primary	Transconjunctival	Autologous calvarial bone graft	Titanium mesh (OsteoMed Co), pre-contoured on patient-specific 3D-printed patient model
Gupta et al	2021	16	23	NR	NR	NR	Primary	Existing scar or subciliary	Porous polyethylene (Biopore™) sheet	Titanium mesh (manufacturer not disclosed) pre-contoured on patient-specific 3D-printed model
Khomutinnikova et al	2020	22	17	NR	NR	NR	Primary	NR	Reperen polymer implants (38.4%), Konment metal implants (30.8%), Synthes implants (30.8%) – further details not disclosed	Individualised polytetrafluroethylene implants (Ecoflon)
Kim et al	2017	38	44	36.5 (16.9)	20:62	IPV 25 Falls 27 Sports 19 RTC 11	Primary	Transconjunctival or transcaruncular	SynPOR titanium mesh (Synthes)	SynPOR PSI titanium mesh (Synthes)
Nkenke et al	2011	10	10	34.9 (13.5)	1:19	NR	Secondary	Subciliary, transconjunctival ± lateral canthotomy	Matrix MIDFACE Preformed orbital plates (Synthes)	CAM/CAM individually shaped adapted glass-bioceramic implants (Bioverit II)
Raisian et al	2017	5	5	34 (11.53)	2:8	RTC 3 Fall 3 Accident 4	Primary	NR	Titanium mesh (manufacturer not disclosed)	Titanium mesh (manufacturer not disclosed) pre-contoured on 3D-printed patient model
Scolozzi et al	2010	10	10	44.8 (19.1)	4:16	NR	Primary	NR	Orbital titanium mesh plate (manufacturer not disclosed)	Pre-formed orbital titanium mesh plates (Synthes)
Sigron et al	2020	12	10	50.1 (18.5)	12:10	IPV 6 Fall 12 Sport-accident 3 Work-injury 1	Primary	Transconjunctival, transcaruncular – approach identical between groups	Matrix MIDFACE Preformed orbital plates (Synthes)	Matrix MIDFACE orbital plates (Synthes) or MODUS Midface OPS 1.5 plates, pre-contoured on patient-specific 3D model
Timoshchuk et al	2022	61	24	38.7 (16.6)	23:62	IPV 54 RTC 9	Primary	NR	Orbital titanium mesh plate (manufacturer not disclosed)	SLM titanium PSI (KLS Martin)
Zimmerer et al	2016	100	95	41.5 (17.0)	48:147	NR	Primary	Transconjunctival or transcutaneous	Matrix MIDFACE Preformed orbital plates (Synthes)	CAD-based: KLS Martin individualised implants; pre-contoured Orbital Floor Mesh Plates (Synthes) Non-CAD-based: Orbital Floor Mesh Plates (Synthes), SynPOR Titanium Reinforced Fan Sheets (Synthes), Stryker MEDPOR Orbital Reconstruction Implants

Sample size was variable between studies, ranging from 5 [21] to 100 [24] per cohort. The weighted mean patient age across all reporting studies was 39.35 (SD 15.72) years, encompassing 550 patients [16, 17, 20–24]. Patients were disproportionately male, accounting for 68% of included cases. Two studies did not provide data on either patient age or sex [18, 19]. Of the 11 identified studies, 10 reported outcomes on primary orbital reconstruction, whereas Nkenke et al. (2011) reported patients undergoing secondary reconstruction (Table 3).

Six of the 11 studies provided some indication of mechanism of injury [16, 17, 21, 23–26]. Interpersonal violence (IPV) was reported in the majority of cases, accounting for 41.7%. Road traffic collisions (RTC) accounted for 21.0%, falls for 18.2%, sports injuries for 11.5%, ‘other’ for 7.3%, and work-related injuries for 0.3%.

**Fig. 2:**
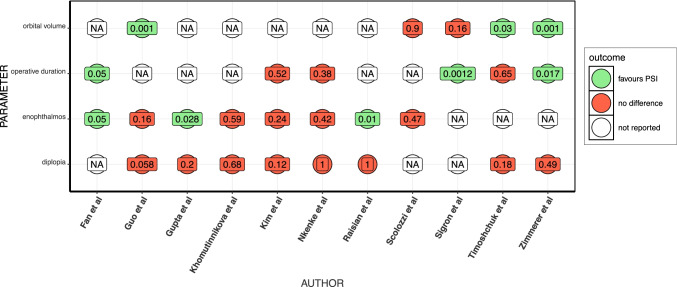
Summary of key outcomes from each paper. Included results include only those where a *p*-value was either reported or could be calculated from the available data. No studies reported significantly improved results in any documented parameter within the conventional group, hence the absence of “favours conventional.” Numbers within each point indicates *p*-value of the comparison between conventional and PSI for the corresponding outcome

Four studies provided specific data on delay to surgery from injury [16, 23, 25, 26], with a pooled weighted mean of 13.11 (SD 29.5; *n* = 137) days in the conventional group compared to 11.2 (SD 19.02; *n* = 107) days in the patient-specific group. Two broad categories were used: either PSI using 3D printing in the fabrication process, usually using an external manufacturer following provision of patient DICOM data; or hybrid-PSI involving pre-operative contouring of conventional implants on patient-specific 3D-printed models. Four studies used solely the hybrid-PSI method [17, 21, 23, 25], whereas 4 studies used CAD/CAM-manufactured PSI only [16, 20, 22, 26] and the remainder used a combination of the two methods. Zimmerer et al. (2016) grouped individualised implants into CAD/CAM-based and non-CAD/CAM-based implants. Here, they have defined CAD/CAM-based implants as those involving both industrial production of PSI using KLS Martin and those implants pre-contoured on a 3D-printed model prior to operation. Conventional methods of reconstruction ranged from standard alloplastic implants (both titanium and porous polyethylene) to autologous calvarial bone grafting. A summary of outcomes from individual outcomes is provided in Fig. 2.

### Follow-up

The follow-up duration and time of data acquisition post-operatively were reported in all studies except two [16, 23]. All reporting studies completed follow-up at multiple time points, ranging from 1 week post-operatively to 5 years. The shortest time point for data acquisition was 12 weeks post-operatively [24] and the longest was 5 years [19].

### Operative duration

**Fig. 3:**
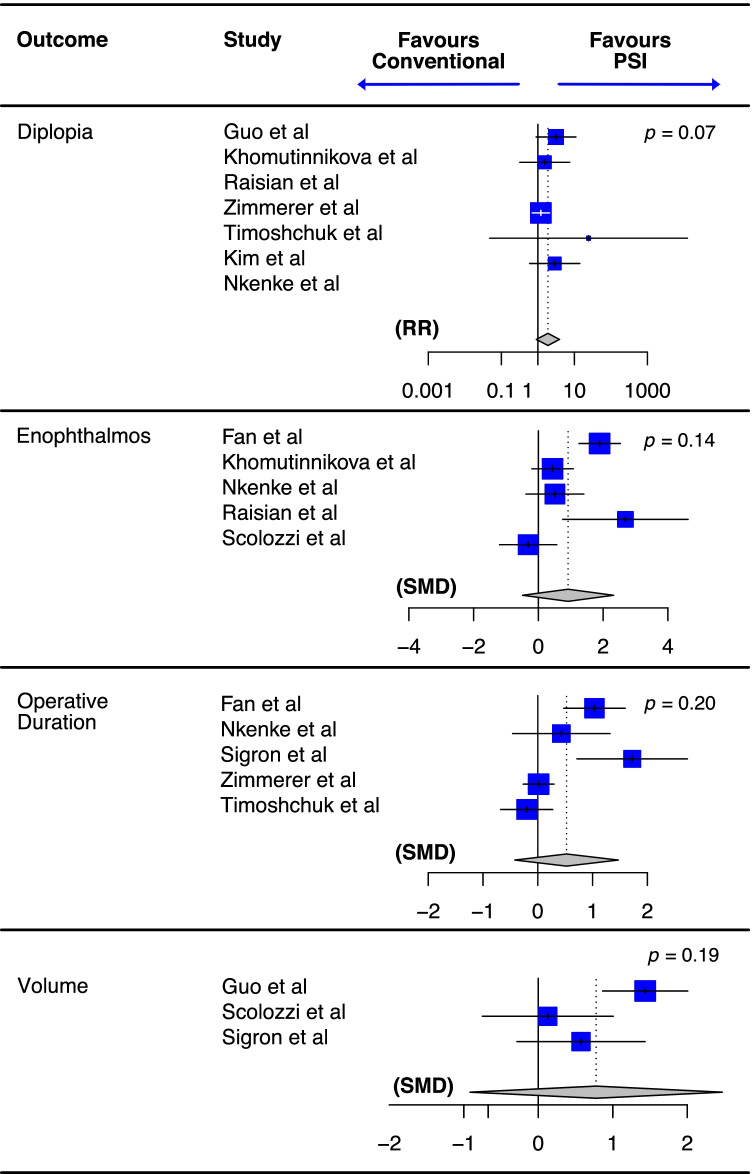
Summary forest plots of key outcomes from reporting studies. RR, relative risk; SMD, standardised mean difference; CI, confidence interval; PSI, patient-specific implant. *p*-values refer to the test for overall effect

Six studies provided data on the duration of orbital reconstruction between patient-specific and conventional implant types [16, 20, 23–26] though Kim et al. provided no indication of variance and so data could not be included in meta-analysis. The weighted mean time for operative duration was 101.3 min (SD 54.08) for patient-specific compared to 101.9 min (SD 62.1) for conventional implants. It should be noted that, in Timoshchuk et al., Fan et al.., and Zimmerer et al., plate bending onto a patient-specific model was performed intra-operatively, thereby limiting the potential time benefit from a pre-shaped patient-individualised plate. Heterogeneity between reporting studies was found to be significant (*Q*(3) = 18.24, *p* < 0.1; *I*^2^ = 83.6%), and no significant difference in operative duration was identified between groups on meta-analysis (SMD = 0.52 [95% CI − 0.42; 1.47], *p* = 0.20; Fig. 3; Supplementary Fig. S1). On an individual study level, the average operative duration was significantly shorter in the patient-specific group vs. the control group in Fan et al. [16] (75.34 min vs. 95.37 min, *p* < 0.05), Sigron et al. [23] (57.30 min vs. 99.80 min, *p* = 0.0012), and Zimmerer et al. [24] (60 min vs. 71 min, *p* = 0.017). No significant difference in mean operative duration was found by Nkenke et al. (2011) (58.5 min and 65.8 min for conventional and control groups, respectively, *p* = 0.38).

### Orbital volume

Only three studies provided volumetric data amenable to pooling between patient-specific and conventional implants [17, 22, 23]. Although Zimmerer et al. (2016) did perform volumetric analysis, it concerned only the variances in orbital volume between the reconstructed and uninjured orbits, as a means to assess precision of reconstruction between the two groups. Thus, volumetric data were reported as range (of volume differences) and variances, with no measure of central tendency and a mean could, thus, not be calculated from the provided data. It is worth noting, however, that precision was statistically better in orbital reconstruction using PSI in this study than with conventional implants (*p* < 0.001) [24]. Similarly, Timoshchuk et al. did perform a volumetric analysis, but volumetric differences were reported as percentage differences and so data was not amenable to pooling. A significantly smaller volume difference between injured and uninjured orbits was identified using patient-specific compared to conventional implants (*p* = 0.03). Of the 3 studies that reported differences between reconstructive volume and uninjured volume, the weighted mean difference was 0.32 ml (SD 0.74; *n* = 55) for PSI compared to 0.95 ml (SD 1.03; *n* = 48) for conventional implants. Heterogeneity was significant (*Q*(2) = 6.87, *p* = 0.03; *I*^2^ = 70.9%), and a random-effects model showed no difference in absolute volume difference (between uninjured orbit and reconstructed orbit) between conventional and PSI (SMD 0.78 [95% CI − 0.91; 2.47], *p* = 0.19; Fig. 3). Guo et al. [17] reported no significant difference in orbital volume between post-operative and unaffected orbits using PSI, but did report a significant difference in those patients undergoing reconstruction using bone, thereby implying better adaptation to premorbid orbital volume with the use of the former. Moreover, re-analysis of weighted pooled absolute volume difference (between operated and unaffected orbits) identified a significantly smaller volume difference in the patient-specific group compared to the conventional group (mean 0.916 ml (SD 0.721) vs. 0.121 ml (SD 0.128), *p* < 0.001). Scolozzi et al. [22] found no significant difference in post-operative orbital volume between conventional and PSI groups (*p* = 0.896). Equally, re-analysis of mean absolute volume differences between post-operative and unaffected orbits showed no statistically significant difference between conventional and PSI (*p* = 0.1617) in Sigron et al. [23]. However, the authors did identify a significant orbital volume difference between pre-operative unaffected orbit and post-operative orbit in the conventional group, but not the patient-specific group, which again might imply better reconstruction with PSI. They conclude that the use of pre-manipulated, patient-specific hybrid titanium meshes can result in at least equally accurate orbital volume reconstruction compared to intra-operative free-hand manipulated titanium mesh.

### Enophthalmos

Five studies reported a specified measurement of post-operative enophthalmos between conventional and patient-specific groups [16, 19–22, 25]. Pooled weighted mean post-operative difference in enophthalmos (between post-operative and unaffected orbits) was 1.39 mm (SD 1.40) for the conventional group compared to 0.55 mm (SD 0.76) for the patient-specific group. Meta-analysis, however, identified no significant difference in enophthalmos between the two groups in these studies (SMD 0.9144 [95% CI − 0.49 to 2.32], *p* = 0.15; *Q*(4) = 22.56, *p* < 0.001; *I*^2^ = 82.3%; Fig. 3). Independently, Fan et al. (*p* = 0.028) (2017) and Raisian et al. (*p* = 0.01) (2017) both found a significant reduction in post-operative enophthalmos in the patient-specific group compared to the control group. Guo et al. (2009) reported the number of patients with post-operative enophthalmos, defined here as a difference in globe projection beyond the lateral orbital margin > 2 mm between the injured and uninjured orbits. Pre-operatively 76.9% of patients in the conventional group and 82.9% in the patient-specific group displayed enophthalmos, reducing to 38.5% and 17.1%, respectively, post-operatively. Re-analysis of data provided by Khomutinnikova et al. (2020), according to the criterion of Guo et al. (2009) (post-operative enophthalmos defined as > 2 mm between uninjured and operated orbit), identified no significant difference in post-operative enophthalmos between groups (*p* = 0.5901). Nkenke et al. (2011) identified tendency towards relapse of enophthalmos in both groups at post-operative day 365 (difference between operated and uninjured orbits of − 0.39 mm (SD 1.06) vs. − 0.07 mm (SD 0.63) for control vs. patient-specific groups); however, the difference in post-operative enophthalmos between groups was not significant (*p* = 0.4226). Gupta et al. (2021) used a patient-specific success score to report on enophthalmos, identifying a statistically significant improvement in enophthalmos in the patient-specific group compared to the conventional group (*p* = 0.028).

### Diplopia

Eight studies reported on the presence of post-operative diplopia [17–21, 24–26]. As with enophthalmos, Gupta et al. (2021) used a success score to study diplopia and found no difference in post-operative diplopia between the group treatment arms (*p* = 0.2). Guo et al. [17] reported an incidence of pre-operative diplopia of 30.8% in the control group, compared to 51.4% in the patient-specific group. This improved to 26.9% in the conventional group vs. 8.6% in the patient-specific group, regardless of fracture age at the time of operation. However, despite this, no significant difference in pre- and post-operative diplopia was identified between the two groups (*p* = 0.058). Only one of the 10 patients reported by Raisian et al. [21] exhibited pre-operative diplopia; a comparison between conventional and control was not possible given complete resolution of diplopia in this patient (and no other cases of diplopia across the studied cohort). Zimmerer et al. [24] found no significant difference between conventional and control groups with respect to post-operative diplopia at 12-week follow-up (*p* = 0.492). Of the 7 studies reporting specific frequencies of post-operative diplopia, diplopia was present in 11.2% (22 of 196 patients) and 19.2% (47 of 245 patients) in patient-specific and conventional groups, respectively. A random-effects model identified a relative risk of diplopia of 1.90 with the use of conventional implants vs. patient-specific, though this did not reach statistical significance (RR 1.90 [95% CI 0.91; 3.92], *p* = 0.07; *Q*(4) = 3.35, *p* = 0.50, *I*^2^ = 0%; Fig. 3).

## Discussion

Despite the prevalence of post-traumatic orbital injuries, there is still a lack of consensus in the literature as to the optimal management pathway for this cohort of patients. This divergence encompasses not only the timing of surgery (i.e. how soon post-injury elective orbital reconstruction should take place), but also the operative approach and the implant-type employed in reconstruction of the orbital architecture [27, 28]. A variety of implant materials and types have been used, including both alloplastic and autologous implants, and resorbable and non-resorbable implants. The choice of implant is influenced by various factors, including fracture complexity and location, operator familiarity and preference, as well as resource availability [29]. The emergence of CAS has accompanied advancements in surgery in a number of ways, from both a pre-operative planning perspective and intra-operative surgical approach. This includes the implementation of stereolithography in creating 3D models of patient anatomy and real-time operative navigation and guidance [30, 31]. Specifically, CAD/CAM processes have enabled an increasing degree of customisation to be afforded to individual patients. With regard to orbital reconstruction, the ability to generate fully customised PSI to accurately replicate the complex and elaborate anatomy of the orbital region is one way in which improvements to pre-existing surgical methods have been attempted. The ability to implement the use of hybrid-PSI, whereby conventional, mass-produced implants are manipulated pre-operatively based on stereolithographically engineered patient models from CT imaging data, represents another potential method of optimising patient outcomes [32]. The hypothetical advantages conferred by PSI (both PSI and hybrid-PSI) include not only the potential for reduced injury-associated complications (diplopia, enophthalmos, reduced visual acuity, etc.) and reduced intra-operative complications, but also the possibility of reduced operating time, obviating the need to manipulate the implant intra-operatively [33].

The purpose of the current study, comprising a total of 628 patients across 11 studies, was to collate relevant comparative data in order to elucidate whether there are any differences in outcomes in patients undergoing orbital reconstruction post-trauma, specifically with regard to patient-specific versus conventional implants. From an epidemiological perspective, the demographic domains of age (mean 39 years) and sex (approximately 2:1 male to female ratio) of the patients were generally similar to data elsewhere in the literature [34]. Likewise, the three most prevalent aetiological reasons for trauma, interpersonal violence, road traffic collisions, and falls, are consistent with the mechanisms of injury most widely reported in the literature [34–36]. Insufficient data were available, however, to determine whether the baseline characteristics between treatment groups were similar between studies.

The benefits of reduced operating time have been described in the literature. It has been suggested that operations of shorter duration are associated with improved clinical outcomes, such as reduced estimated blood loss, and reduced overall length of hospital admission [33]. Moreover, efficient operating can have subsequent ramifications on an organisation’s overall productivity [37]. Although not supported by meta-analysis, 3 of the 6 reporting studies independently identified significantly shorter operative duration in the group of patients undergoing reconstruction with PSI, supporting the notion of more rapid implant placement.

Similarly to operative duration, weighted post-operative mean difference in orbital volume between the post-operative orbit and contra-lateral unaffected orbit was smaller in the patient-specific group, indicating better reconstitution of pre-operative orbital anatomy. Despite non-significance on meta-analysis, it is telling that 3 of the 4 studies reporting on changes in orbital volume identified significantly better outcomes with the use of PSI, whether by virtue of a reduction in the orbital volume difference between affected and unaffected orbits, or as a result of the degree of precision in the reconstruction. Three of the 8 reporting studies also found a statistically significant benefit of PSI in improving post-operative enophthalmos, though a commensurate improvement was apparently not translated to diplopia (Fig. 3).

Two of the 11 papers eligible for inclusion reported on patient randomisation between treatment arms; however, the method of randomisation was not made explicit [18, 21]. Failure to account for baseline differences in patient characteristics between groups using a robust process of randomisation will introduce bias. Moreover, outcome measurements on post-operative imaging were not blinded. Although this is not feasible where the control group implant is obviously distinct from the PSI (in the case of autologous bone, for example), blinding may be possible, at least to the individual interpreting post-operative imaging and to the patient, with the use of conventional versus hybrid-patient-specific plates. Baseline characteristics that might introduce heterogeneity include the fracture characteristics (severity or complexity, pattern, location); implant types used (alloplastic and autologous implants in the conventional groups); implant materials used (titanium and porous polyethylene in the conventional groups), degree of customisation employed (PSI or hybrid-PSI); operative approach to reconstruction; time to surgery from injury; and type of reconstruction (one study evaluated secondary reconstruction whilst the remainder examined primary reconstruction). Moreover, important clinical outcomes, such as hypoglobus, ION hypoaesthesia, ocular motility restriction, and procedure-related complications, could not be included in the current study due to insufficient reporting. A recent systematic review assessing patient-specific vs. conventional titanium mesh implants in post-traumatic orbital reconstruction, Hartmann et al. (2021), drew similar conclusions to the present study with generally positive outcomes across a range of clinical domains being attributed to the patient-specific group [12]. Nevertheless, Hartmann et al. also reported encountering significant heterogeneity for various reasons including study design, materials, and non-standardised reporting. The present study expands on the results of this previous review, identifying a further 9 comparative studies that assess outcomes directly between patient-specific and conventional implants for traumatic orbital reconstruction.

A potential limitation of the current study is the pooling of patient-specific and hybrid-patient-specific plates as a single comparator group encompassing PSI. Despite the similarities between these two subgroups, the authors acknowledge that there are intrinsic differences relating to the different manufacturing processes, namely that hybrid-patient-specific plates (by virtue of modification and manipulation from conventional, mass-produced plates) will likely have sharper edges than plates of the patient-specific cohort and assumed less accurate adaptation to the printed 3D model relative to patient-specific plates. Moreover, in three of the included studies, manipulation of hybrid-patient-specific plates was performed intra-operatively, thereby negating any potential advantage on operative duration of pre-operatively contoured PSI [16, 24, 26]. Collectively, these differences may have a discernible impact on intra-operative placement and post-operative complications. Nonetheless, there are several parallels between these two subgroups: both methods not only utilise CAM/CAM technology and MIO for virtual reconstruction, but also employ stereolithography at some point in the manufacturing process. Fundamentally, both patient-specific and hybrid-patient-specific manufacturing methods result in the application of an individualised implant at the time of surgery.

A further consideration with patient-individualised therapies, irrespective of disciplines, is that they are initially expensive. A study on cost-effectiveness of patient-specific versus conventional implants is beyond the scope of the current review. However, a previous review on costs of conventional implants in orbital reconstruction found that the mean cost of the implant itself ranged from $70.25 to $7718.00, with a mean total cost of care per patient of $35,585,57, the majority of which was attributable to theatre running costs, inpatient stay, and clinical review [38]. The manufacturer cost of PSI involving the use of CAD/CAM is undoubtedly higher than that of conventional implants. Given the potential for shorter operative duration, shorter inpatient admission, and fewer post-operative complications, however, this unit cost deficit may well be offset.

## Conclusion

Although some individual studies reported a potential advantage of patient-specific implants for reducing operative time, improved orbital volume recapitulation, and better outcomes with respect to post-operative enophthalmos, statistically significant results were not demonstrated on meta-analysis to reflect this. There are several potential reasons for this, which likely relate to the retrospective nature of the identified observational cohort studies with accompanying lack of randomisation. Inevitably there will be differences in fracture complexity, operator experience and technique, as well as patient baseline demographics between groups, which may limit the magnitude of detectable outcome differences attributable to treatment effects. The use of CAD/CAM is still a relatively emergent technology within the oral and maxillofacial surgery domain. As these technologies become more widely adopted within the community, it is the authors’ opinion that the benefits of this technology will be fully realised. Based on the results of this study, the choice of implant used should, thus, be left to the discretion of the surgeon.

## Supplementary Information

Below is the link to the electronic supplementary material.

Supplementary file 1 Supplementary Fig. S1: Complete forest plots, including raw data used in random-effects models. (PDF 57 KB)

Supplementary file 2 (PDF 96 KB)
