# Supplementary material for: Orbital reconstruction: a systematic review and meta-analysis evaluating the role of patient-specific implants
Source: Oral Maxillofac Surg. 2022 May 20;27(2):213–26. doi: 10.1007/s10006-022-01074-x (PMC10234907; doi:10.1007/s10006-022-01074-x)

DIPLOPIA

Study	Conventional Diplopia	Conventional Total	PSI Diplopia	PSI Total	RR	95%-CI	Weight
Guo et al	7	26	3	35	3.14	[0.90;11.00]	22.4%
Khomutinnikova et al	4	22	2	17	1.55	[0.32;7.47]	16.7%
Raisian et al	0	5	0	5			0.0%
Zimmerer et al	25	84	15	61	1.21	[0.70;2.10]	42.9%
Timoshchuk et al	6	60	0	24	24.46	[0.05; 12428.25]	1.5%
Kim et al	5	38	2	44	2.89	[0.60;14.07]	16.6%
Nkenke et al	0	10	0	10			0.0%
**Random effects model**		**245**		**196**	**1.89**	**[0.91; 3.92]**	**100.0%**

Heterogeneity: $I^2 = 0\%$, $\tau^2 = 0.2825$, $p = 0.50$

Test for overall effect: $t_4 = 2.41$ ($p = 0.07$)

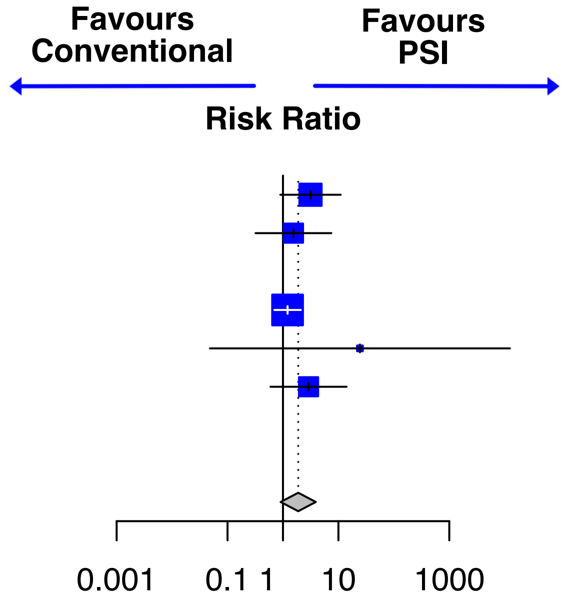

ENOPHTHALMOS

Study	Conventional Total	Conventional Mean	Conventional SD	PSI Total	PSI Mean	PSI SD	SMD	95%-CI	Weight
Fan et al	27	2.50	1.0000	29	1.00	0.5000	1.89	[1.25; 2.53]	22.7%
Khomutinnikova et al	22	0.95	1.2400	17	0.46	0.8700	0.44	[−0.20; 1.08]	22.6%
Nkenke et al	10	−0.07	0.6300	10	−0.39	0.5700	0.51	[−0.38; 1.40]	20.8%
Raisian et al	5	2.40	0.8900	5	0.30	0.4500	2.69	[0.75; 4.62]	13.0%
Scolozzi et al	10	0.30	0.4800	10	0.50	0.7100	−0.32	[−1.20; 0.57]	20.9%
**Random effects model**	**74**			**71**			**0.92**	**[−0.49; 2.32]**	**100.0%**

Heterogeneity: $I^2 = 82\%$, $t^2 = 1.0606$, $p < 0.01$

Test for overall effect: $t_4 = 1.81$ ($p = 0.14$)

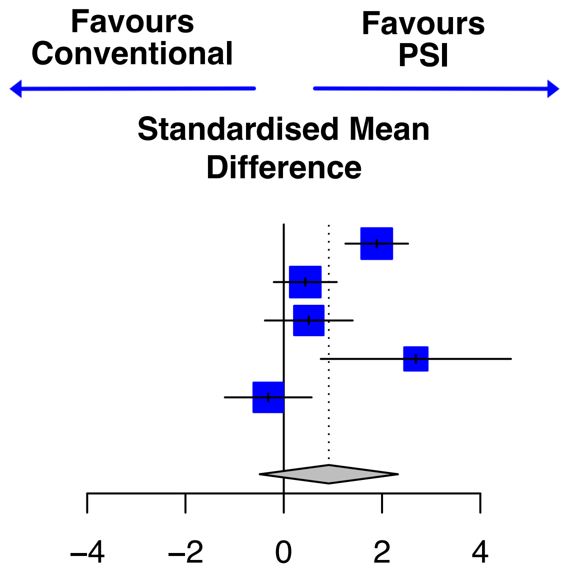

OPERATIVE DURATION

Study	Conventional Total	Conventional Mean	Conventional SD	PSI Total	PSI Mean	PSI SD	SMD	95%-CI	Weight
Fan et al	27	95.37	22.1900	29	75.34	15.6800	1.03	[0.47; 1.59]	21.1%
Nkenke et al	10	65.80	14.6000	10	58.50	17.8000	0.43	[−0.46; 1.32]	17.3%
Sigron et al	12	99.80	23.9000	10	57.30	23.5000	1.72	[0.72; 2.73]	15.9%
Zimmerer et al	100	127.25	61.4300	95	126.25	69.5500	0.02	[−0.27; 0.30]	23.7%
Timoshchuk et al	60	67.00	32.5000	24	74.30	42.5000	−0.20	[−0.68; 0.27]	22.0%
**Random effects model**	**209**			**168**			**0.52**	**[−0.42; 1.47]**	**100.0%**

Heterogeneity: $I^2 = 82\%$, $t^2 = 0.4719$, $p < 0.01$

Test for overall effect: $t_4 = 1.54$ ($p = 0.20$)

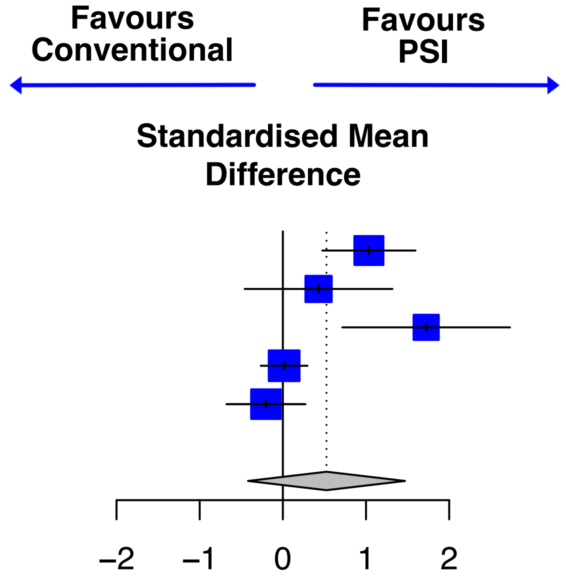

ORBITAL VOLUME

Study	Conventional Total	Conventional Mean	Conventional SD	PSI Total	PSI Mean	PSI SD	SMD	95%-CI	Weight
Guo et al	26	0.92	0.7310	35	0.19	0.2000	1.43	[0.86; 2.01]	39.1%
Scolozzi et al	10	0.26	1.0716	10	0.08	1.5682	0.13	[−0.75; 1.01]	30.2%
Sigron et al	12	1.60	1.2000	10	1.00	0.7000	0.57	[−0.29; 1.43]	30.7%
**Random effects model**	**48**			**55**			**0.78**	**[−0.92; 2.47]**	**100.0%**

Heterogeneity: $I^2 = 71\%$, $t^2 = 0.3059$, $p = 0.03$

Test for overall effect: $t_2 = 1.97$ ($p = 0.19$)

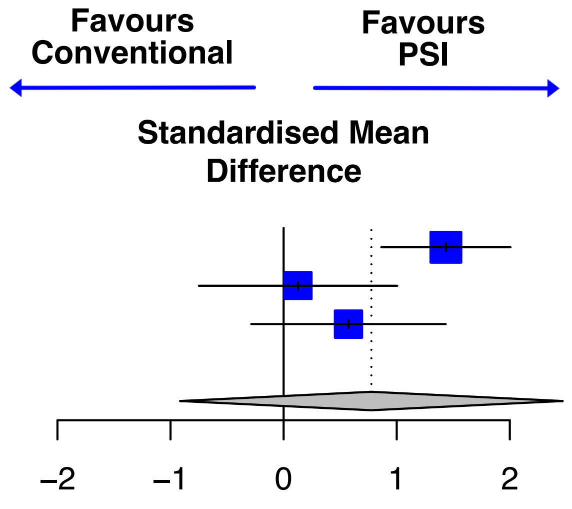

Supplement: Supplementary file 1 — Supplementary Fig. S1: Complete forest plots, including raw data used in random-effects models. (PDF 57 KB) [file 10006_2022_1074_MOESM1_ESM.pdf]
