# Supplementary material for: Orbital reconstruction: a systematic review and meta-analysis evaluating the role of patient-specific implants
Source: Oral Maxillofac Surg. 2022 May 20;27(2):213–26. doi: 10.1007/s10006-022-01074-x (PMC10234907; doi:10.1007/s10006-022-01074-x)
Supplement: Supplementary file 2 — (PDF 96 KB) [file 10006_2022_1074_MOESM2_ESM.pdf]

SUPPLEMENTARY MATERIAL

Search strategy for included databases

• ClinicalTrials.gov (n = 18)

1. Orbital Fracture

• Cochrane CENTRAL (n = 120)

1. Orbital Fracture

• EMBASE and MEDLINE (via OVID) (n = 356 and 1227, respectively)

1. exp Orbital Fractures/
2. exp Orbital Implants/
3. exp Maxillofacial Injuries/
4. exp Reconstructive Surgical Procedures/
5. orbital reconstruct$.mp.
6. orbital fracture$.mp.1W
7. 1 or 2 or 3 or 4 or 5 or 6
8. exp Surgery, Computer-Assisted/
9. exp Therapy, Computer-Assisted/
10. exp Diagnosis, Computer-Assisted/
11. exp Computer-Aided Design/
12. exp Stereolithography/
13. exp Computer Simulation/
14. exp Imaging, Three-Dimensional/
15. exp Tomography, X-Ray Computed/
16. preform$.mp.
17. prefabricate$.mp.
18. exp Stereotaxic Techniques/
19. exp Neuronavigation/
20. 8 or 9 or 10 or 11 or 12 or 13 or 14 or 15 or 16 or 17 or 18 or 19
21. exp Randomized Controlled Trial/
22. trial.ab,ti.
23. cohort.ab,ti.
24. 21 or 22 or 23
25. *7 and 20 and 24*

• PubMed (n = 685)

1. (((((orbital fracture) OR (orbital implant)) OR (orbital reconstruction)) AND (((((((((((3D) OR (stereolithography)) OR (CT)) OR (computer-assisted)) OR (computer-aided)) OR (computer-guided)) OR (preformed)) OR (prefabricated)) OR (3D imaging)) OR (stereotactic)) OR (stereotaxic)) OR (neuronavigation))) AND (((randomized controlled trial) OR (cohort)) OR (trial))

• Scopus (n = 1896)

1. (KEY(orbital AND fracture) OR KEY(orbital AND implant) OR KEY(orbital AND reconstruction) AND KEY(3D) OR KEY(computer-assisted) OR KEY(computer-aided) OR KEY(computer-guided) OR KEY(stereotactic) OR KEY(stereotaxic) OR KEY(stereolithography) OR KEY(CT) OR KEY(preformed) OR KEY(prefabricated)

- WOSCC (n = 461)

    0. "TS=Orbit* Fracture*
    1. TS=Orbit* Reconstruct*
    2. TS=Orbit* Implant*
    3. TS=Maxillofacial Injuries
    4. 1 or 2 or 3 or 4
    5. TS=Computer-Aided
    6. TS=Computer-Guided
    7. TS=Computer-Assisted
    8. TS=3D
    9. TS=CT
    10. TS=Preformed
    11. TS=Prefabricated
    12. TS=Stereotactic
    13. TS=Stereotaxic
    14. TS=Neuronavigation
    15. 8 or 9 or 10 or 11 or 12 or 13 or 14 or 15
    16. TS=Randomized Controlled Trial
    17. TS=Trial
    18. TS=Cohort
    19. TS=Control
    20. TS=Groups
    21. 17 or 18 or 19 or 20 or 21
    22. 5 and 16 and 22"

- WHO ICTRP (n = 21)

    1. "Orbital Fracture"
